# Selective AUTAC‐mediated ATP7B degradation unlocks cuproptosis vulnerability in pancreatic ductal adenocarcinoma

**DOI:** 10.1002/ctm2.70848

**Published:** 2026-10-27

**Authors:** Zihan Zhang, Min Li, Jiale Feng, Jian‐Ang Li, Lei Zhang

**Affiliations:** ^1^ Department of Interventional Radiology Zhongshan Hospital Fudan University Shanghai China; ^2^ Department of Pancreatic Surgery Zhongshan Hospital Fudan University Shanghai China

**Keywords:** ATP7B, cuproptosis, drug development, pancreatic ductal adenocarcinoma, targeted therapies

## Abstract

Cuproptosis arises from mitochondrial copper overload, yet how tumour cells evade this metabolic vulnerability remains unclear. We identify ATP7B as the central barrier to cuproptosis in pancreatic ductal adenocarcinoma (PDAC). ATP7B is aberrantly upregulated in PDAC and predicts poor prognosis. Its depletion increases intracellular copper, activates FDX1, DLAT/DLST lipoylation, disrupts tricarboxylic acid (TCA) metabolism, and reinstates cuproptosis in vitro and in vivo. Mechanistically, ATP7B undergoes ZBTB10‐driven K63‐linked ubiquitination, recognized by NBR1 for selective autophagic degradation. Leveraging this, we developed an ATP7B‐targeting autophagy‐targeting chimera (ATAUTAC) that promotes ATP7B clearance, amplifies copper‐driven mitochondrial metabolic perturbations, triggers cuproptosis, and inhibits tumour growth with favourable tolerability. Notably, ATAUTAC sensitizes PDAC to gemcitabine, overcoming resistance by exposing latent metabolic fragility. These findings establish ATP7B as a mechanistic hub of cuproptosis tolerance and demonstrate that autophagy‐based ATP7B degradation is a druggable strategy for copper‐dependent tumour eradication. ATAUTAC offers a promising avenue for precision therapy in PDAC and other refractory malignancies.

## Key points

- ATP7B is markedly upregulated in pancreatic ductal adenocarcinoma.
- ATAUTAC selectively degrades ATP7B via autophagy‐dependent targeting.
- ATAUTAC enhances copper‐induced stress and suppresses PDAC tumour progression.

## Graphical abstract

ATP7B is aberrantly upregulated in PDAC cells, contributing to the maintenance of copper homeostasis. We designed ATAUTAC, a targeted autophagy‐dependent degradation molecule that selectively eliminates ATP7B. By disrupting copper balance, ATAUTAC sensitizes PDAC cells to cuproptosis, thereby amplifying copper‐induced metabolic stress and effectively inhibiting tumour progression.

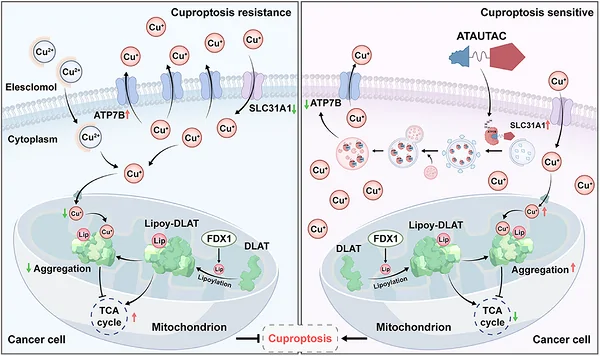

## 1 INTRODUCTION

Metal‐ion homeostasis is essential for sustaining cellular viability and coordinating key regulatory processes.^1^, ^2^ Many malignancies, including pancreatic ductal adenocarcinoma (PDAC), exhibit a markedly increased demand for metal ions during rapid proliferation, which drives a reprogramming of iron and copper uptake and utilization to maintain elevated intracellular metal loads.^3^, ^4^, ^5^ Based on this metabolic vulnerability, the recently proposed cell‐death modality termed cuproptosis reveals a distinctive mechanism in which copper binds to lipoylated proteins within the tricarboxylic acid (TCA) cycle, ultimately causing mitochondrial collapse and cell death.^6^ This discovery provides an important therapeutic opportunity for targeting metal‐ion‐dependent metabolic rewiring in cancer.

While several pro‐cuproptosis strategies have been developed, most currently rely on direct copper ionophores or natural products to force massive, non‐specific intracellular copper loading.^7^ However, these approaches often suffer from poor tumour selectivity and cause severe systemic heavy‐metal toxicity in healthy tissues.^8^ Furthermore, tumour cells often acquire tolerance by strengthening their antioxidant capacity or accelerating copper efflux.^9^, ^10^ Among these adaptive mechanisms, Cu‐ATPases such as copper‐transporting ATPase 1 (ATP7A) and copper‐transporting ATPase 2 (ATP7B) serve critical functions in copper loading, vesicular trafficking, and exocytotic export, and they represent major determinants of cellular copper homeostasis as well as key contributors to cuproptosis resistance. In particular, abnormal ATP7B expression facilitates copper efflux and has also been strongly linked to drug resistance.^10^, ^11^ Various strategies targeting ATP7B have been explored, including clinical copper chelators, upstream transcriptional/epigenetic modulators,^12^, ^13^ and occupancy‐driven small‐molecule inhibitors.^14^ However, these approaches are heavily limited. Copper chelators only sequester free ions rather than eliminating the efflux pump, while transcriptional and competitive inhibitors often suffer from transient efficacy, poor target selectivity between ATP7A and ATP7B, or compensatory accumulation of the targeted transport proteins. Consequently, a direct, target‐specific, and pharmacologically tractable method to selectively eliminate the physical ATP7B protein remains an unresolved challenge.

With the rapid advancement of targeted protein degradation technologies, the autophagy pathway has emerged as an important therapeutic platform following the proteasome, largely because of its ability to process large protein complexes and structurally intricate transmembrane substrates.^15^, ^16^, ^17^ Innovative modalities such as autophagy‐tethering compound (ATTEC), autophagy‐targeting chimera (AUTAC), and autophagy‐targeting chimera for targeted protein degradation (AUTOTAC) have enabled efficient clearance of previously “undruggable” macromolecules and have considerably expanded the functional scope of targeted degradation.^18^, ^19^ However, current autophagy‐based degradation approaches predominantly focus on soluble cytosolic proteins or cell‐surface receptors,^20^, ^21^ and effective methods for selectively eliminating transmembrane metal transporters within the endomembrane system, including Cu‐ATPases, are still lacking. Moreover, although autophagy has been extensively implicated in iron homeostasis and ferroptosis regulation, the autophagic mechanisms governing copper efflux remain largely unexplored. Consequently, there is a pressing need to develop an autophagy‐based tool that can selectively eliminate ATP7B, thereby regulating copper homeostasis at its source and reversing resistance to cuproptosis.

In this study, we developed an ATP7B‐targeting autophagy‐targeting chimera (ATAUTAC), the first autophagy‐tethering compound specifically designed to target ATP7B. We demonstrate that ATAUTAC induces potent and selective degradation of ATP7B through the autophagy–lysosome pathway, which substantially enhances tumour‐cell sensitivity to cuproptosis. In both in vitro and in vivo PDAC models, ATAUTAC exhibits strong antitumour efficacy together with an excellent safety profile.

## 2 METHODS

### 2.1 Bioinformatic analysis

Public transcriptomic datasets, including GSE62165, GSE57495, and GSE17891, were retrieved from the Gene Expression Omnibus (GEO) database. PDAC patient cohort data were obtained from The Cancer Genome Atlas (TCGA‐PAAD) database. Standard processing, including background correction, quantile normalization (RMA), and batch effect removal (ComBat), was performed in R. Differential gene expression between PDAC and normal pancreatic tissues was analyzed using the limma package (|log_2_FC| > 1, adjusted *p* < .05). Prognostic values of ATP7B (in GSE57495) and ZBTB10 (in TCGA‐PAAD) were evaluated using Kaplan–Meier survival curves with log‐rank tests. Correlation analyses among cuproptosis signatures, autophagy activity, ZBTB10, and ATP7B levels across the datasets were assessed by Spearman correlation analysis. All visualizations were conducted with *ggplot2* and *pheatmap* packages in R.

### 2.2 Cell culture and transfection

The human pancreatic ductal adenocarcinoma cell lines PANC‐1 [RRID: CVCL_0480], ASPC‐1 [RRID: CVCL_0152], and Patu‐8988t [RRID: CVCL_1847], as well as the normal pancreatic duct epithelial cell line hTERT‐HPNE [RRID: CVCL_C466], were obtained from iCell Bioscience Inc. (Shanghai, China). PANC‐1, ASPC‐1 and Patu‐8988t cells were maintained in DMEM supplemented with 10% FBS and 1% P/S. hTERT‐HPNE cells were cultured in DMEM/F12 medium supplemented with 10% FBS, 5.5 mM glucose and 1% penicillin–streptomycin. All cell lines were cultured at 37°C in a humidified atmosphere containing 5% CO_2_, routinely authenticated by the supplier, and confirmed to be free of mycoplasma contamination in our laboratory. Cells were passaged at 70–80% confluence.

Transient transfection was performed using Lipofectamine 3000 (Invitrogen) according to the manufacturer's instructions. For ZBTB10 overexpression, the pcDNA3.1‐Flag‐ZBTB10 plasmid and corresponding empty vector were constructed by Generay Biotech (Shanghai, China). Briefly, cells were seeded to reach 50–60% confluence and transfected with 2.5 µg of plasmids or siRNAs diluted in Opti‐MEM. After 6 h, the transfection medium was replaced with fresh complete culture medium, and cells were harvested 24–48 h later for analysis. For stable knockdown, lentiviral vectors harbouring shRNAs targeting ATP7B, ATP7A, and FDX1 were packaged and used to infect cells in the presence of 8 µg/mL polybrene, followed by puromycin selection to generate stable cell lines. Knockdown efficiency was confirmed by western blotting.

### 2.3 Detection of intracellular Cu^2+^ levels

Intracellular Cu^2+^ levels were measured using the cell copper (Cu^2+^) colourimetric assay kit (complexing method) (E‐BC‐K775‐M, Elabscience, China). Briefly, cells were harvested, lysed on ice, and centrifuged to obtain supernatants. Samples and standards were incubated with the complexing reagent according to the manufacturer's instructions, and absorbance was measured at 575–585 nm using a microplate reader (BioTek). Cu^2+^ concentrations were calculated from the standard curve. To account for variations in cell density and biomass, the total protein concentration of each sample supernatant was determined using a BCA protein assay kit (Beyotime, China). The raw copper concentration of each sample was normalized to its respective total protein concentration, and the final data were presented as relative levels.

### 2.4 Copper salt staining

Intracellular copper salt was detected using a commercial copper salt stain kit (dithiooxamide method) (G3040, Solarbio, China). Cells grown on coverslips were fixed with 4% paraformaldehyde and incubated with the Dithiooxamide working solution according to the manufacturer's instructions. Images were acquired using a fluorescence microscope (Leica).

### 2.5 Molecular docking analysis

Molecular docking between ATP7B–NBR1 and ATP7B–ZBTB10 was performed using the AlphaFold2 Multimer algorithm. Predicted protein structures were selected based on high pLDDT and ipTM scores, followed by interface evaluation and interaction visualization in PyMOL and Discovery Studio. Docking poses with optimal interfacial complementarity were regarded as the most reliable binding conformations.

### 2.6 Animal experiment

All mice were acclimatized for 1 week before the experiments and housed in a specific‐pathogen‐free (SPF) facility under a strictly controlled environment (temperature: 22 ± 2°C, relative humidity: 50–60%, and a 12 h light/dark cycle) with free access to sterile water and standard rodent chow. Female BALB/c nude mice (4–6 weeks old) were subcutaneously inoculated with ASPC‐1 cells (1 × 10^6^ in 100 µL PBS) into the right flank on day 0. When tumour volume reached approximately 80 mm^3^ (day 5), mice were randomly assigned to the indicated treatment groups. For ATP7B‐interference studies, xenografts generated from shCtrl‐ or shATP7B‐expressing cells received intratumoral administration of Elesclomol (ES, 100 mg/kg) or vehicle every 2–3 days. For ATAUTAC dose evaluation, mice were treated intratumorally with vehicle, 20 mg/kg, or 40 mg/kg ATAUTAC using the same schedule. For combination therapy, mice were randomized into PBS, gemcitabine (GEM), ATAUTAC, or GEM+ATAUTAC groups; GEM was dissolved in saline and administered intraperitoneally at 40 mg/kg on days 5, 7, and 9 and thereafter every 2 days for 14 days, while ATAUTAC was dissolved in a vehicle of 10% DMSO, 40% PEG300, 5% Tween‐80, and 45% saline, and administered intratumorally at 40 mg/kg on the same schedule. Tumour size and body weight were monitored every other day, and tumour volume was calculated as (length × width^2^)/2. At treatment completion, mice were sacrificed, and blood, tumours, and major organs (heart, liver, spleen, lung, kidney) were collected for subsequent histological and biochemical analyses. All animal procedures were approved by the Ethical Approval for Research Involving Animals of Zhongshan Hospital, Fudan University (2025‐02‐19).

### 2.7 Statistical analysis

All data are presented as mean ± standard deviation (SD) from at least three independent experiments. Statistical analyses were performed using GraphPad Prism (version 10.1.2). Two‐group comparisons were conducted using unpaired two‐tailed Student's *t*‐tests, while multiple‐group comparisons were analyzed by one‐way ANOVA followed by Tukey's post hoc test. *p*‐values < .05 were considered statistically significant.

## 3 RESULTS

### 3.1 Silencing ATP7B enhances cuproptosis sensitivity and suppresses PDAC cell growth in vitro and in vivo

Mining the GSE62165 dataset indicated that ATP7B expression is markedly elevated in PDAC compared with normal pancreatic tissues (Figure 1A). Kaplan–Meier analysis further indicated that patients with high ATP7B expression exhibit significantly poorer survival outcomes in PDAC (Figure S1A). Consistently, paired clinical specimen analyses confirmed that both ATP7B mRNA (Figure S1B) and protein levels (Figure 1B) are elevated in PDAC tissues compared with non‐malignant adjacent counterparts. Furthermore, ATP7B expression remained stably elevated across different pathological grades (Figure S1C), clinical stages (Figure S1D), treatment responses (Figure S14E), age groups (Figure S1F), and genders (Figure S1G), demonstrating that ATP7B is a ubiquitous and stable therapeutic target throughout PDAC progression. At the cellular level, ATP7B protein was barely detectable in immortalized human pancreatic ductal epithelial cells (hTERT‐HPNE), whereas PDAC cell lines showed elevated expression, with Patu‐8988t and ASPC‐1 cells exhibiting the highest baseline levels (Figure 1C). To determine whether copper accumulation directly regulates ATP7B expression, Patu‐8988t cells were treated with copper chloride (CuCl_2_). While low concentrations of CuCl_2_ did not compromise cell viability (Figure S1H), copper exposure induced a concentration‐dependent upregulation of both ATP7B protein (Figure S1I) and mRNA levels (Figure S1J), suggesting that ATP7B serves as a responsive copper‐clearance mediator.

**FIGURE 1:**
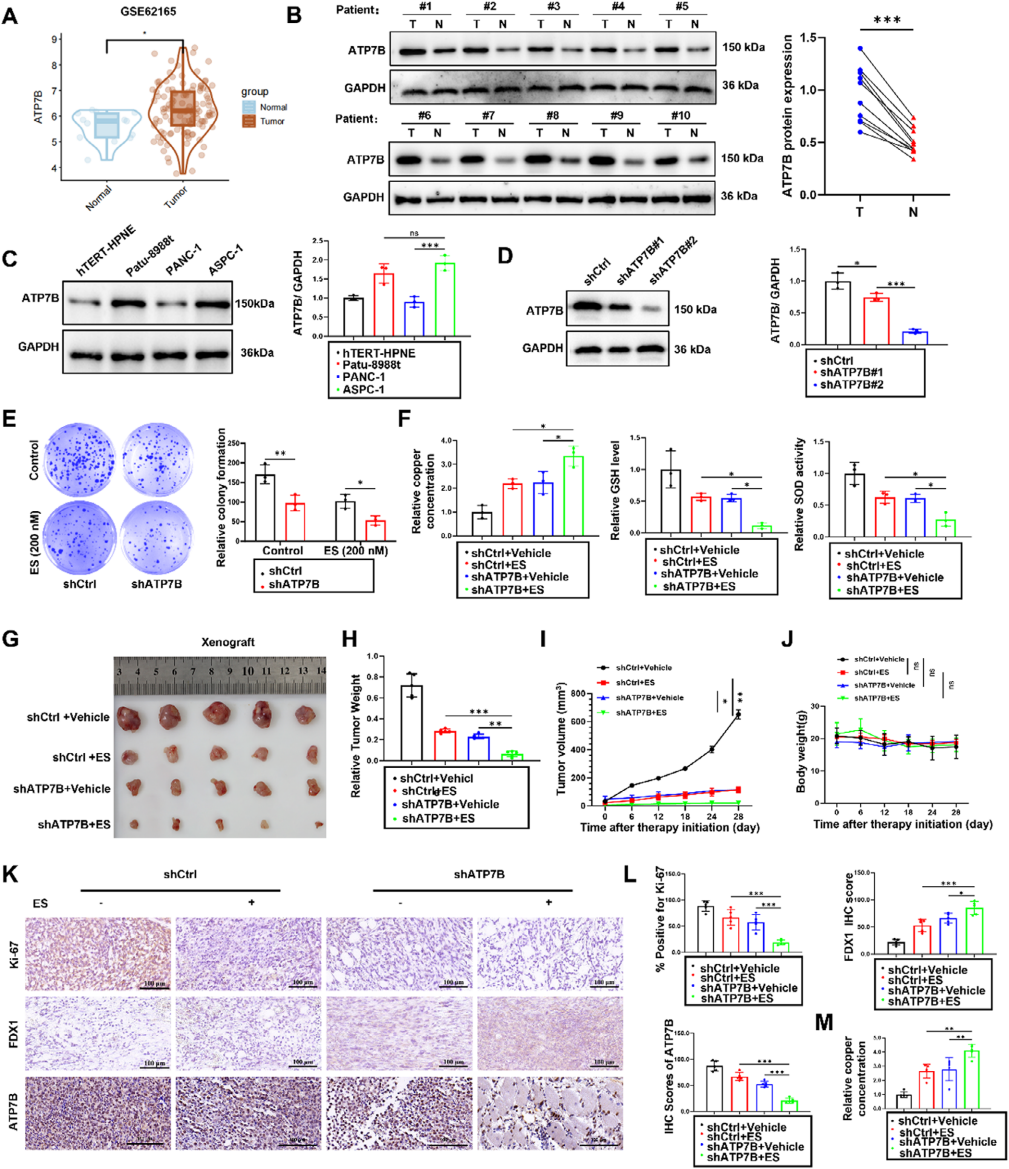
**ATP7B upregulation protects PDAC from cuproptosis**. (A) Analysis of ATP7B transcript levels in normal pancreatic tissues and PDAC tissues from the GSE62165 dataset. (B) Western blot analysis of ATP7B protein in paired tumor (T) and adjacent normal (N) pancreatic tissues from ten PDAC patients, with GAPDH as a loading control. Quantification of relative ATP7B protein abundance is shown on the right. (C) ATP7B protein expression in human pancreatic ductal epithelial cells (hTERT‐HPNE) and PDAC cell lines (PANC‐1, Patu‐8988t, and ASPC‐1). (D) Western blot validation of ATP7B knockdown using shRNA constructs in ASPC‐1 cells. (E) Representative colony formation assay in ATP7B‐silenced and control ASPC‐1 cells treated with vehicle or elesclomol (ES, 200 nM) for 24 h. (F) Relative levels of intracellular Cu^2^^+^, reduced glutathione (GSH), and superoxide dismutase (SOD) activity in ASPC‐1 cells treated with Elesclomol (ES, 200 nM) or vehicle for 24 h. (G–M) Xenografts generated from shCtrl‐ or shATP7B‐expressing ASPC‐1 cells in female BALB/c nude mice received intratumoral administration of Elesclomol (ES, 100 mg/kg) or vehicle (saline) every 2–3 days for 28 days (*n* = 5 mice per group). (G) Gross images of xenograft tumours derived from ATP7B‐silenced or control ASPC‐1 cells, with or without ES administration. (H) Measurement of tumour weight at endpoint in xenograft mice from the indicated experimental groups (*n* = 5 mice per group). (I) Tumour growth curves of xenografts established with control or ATP7B‐depleted ASPC‐1 cells under vehicle or ES treatment (*n* = 5 mice per group). (J) Body weight monitoring of xenograft‐bearing mice during therapeutic intervention (*n* = 5 mice per group). (K) Representative immunohistochemical staining of Ki‐67, FDX1, and ATP7B in xenograft tumours from each treatment group. Scale bar: 100 µm. (L) Quantitative scoring of Ki‐67, ATP7B and FDX1 immunohistochemistry in corresponding xenograft specimens (*n* = 5 mice per group). (M) Relative levels of intratumoral Cu^2^^+^ levels in xenograft tissues from the indicated treatment groups (*n* = 5 mice per group). Data are presented as mean ± SD. *p < .05, **p < .01, ***p < .001; ns, not significant.

To explore the functional roles of ATP7B, ASPC‐1 cells with stable ATP7B silencing were generated (Figure 1D). Loss of ATP7B profoundly reshaped copper homeostasis, rendering PDAC cells substantially more susceptible to cuproptosis. Specifically, the ATP7B‐deficient cells displayed reduced viability and proliferative capacity, elevated intracellular copper accumulation, and diminished antioxidant capacity, collectively reflecting a heightened vulnerability to copper‐induced metabolic stress (Figure S2A,B; Figure 1E,F). We also observed a marked accumulation of high‐molecular‐weight DLAT oligomers in ATP7B‐silenced cells upon copper stress (Figure S2C), which coincided with a distinct punctate aggregation and reorganization of DLAT in the cytoplasm (Figure S2D,E). To distinguish the role of ATP7B from its homolog ATP7A, single and joint knockdowns were performed in Patu‐8988t cells (Figure S2F). ATP7B depletion, but not ATP7A depletion, led to a marked upregulation of FDX1, increased SLC31A1 levels, and elevated DLAT and DLST lipoylation, confirming ATP7B as the dominant copper efflux transporter shielding PDAC cells.

The tumour‐suppressive effect of ATP7B depletion was subsequently recapitulated in vivo using an ASPC‐1 xenograft model. Mice bearing shCtrl or shATP7B tumours were treated with vehicle or ES. Xenografts in the shATP7B+ES group grew significantly slower and exhibited a substantially reduced tumour burden compared with the other cohorts (Figure 1G–I), with no adverse impact on overall body weight (Figure 1J). Histological examination of the tumour tissues demonstrated a reduced percentage of Ki‐67 positive cells, lower ATP7B abundance, and elevated FDX1 levels in the dual‐treatment group (Figure 1K,L). Furthermore, biochemical quantification showed that ATP7B‐deficient xenografts accumulated higher levels of intratumoral copper, which was further elevated upon ES treatment (Figure 1M), reinforcing the role of ATP7B as an essential copper‐buffering mediator in vivo. Collectively, these results suggest that ATP7B serves as a functional driver of cuproptosis tolerance in PDAC, presenting a potential therapeutic target.

### 3.2 Autophagy activation accelerates ATP7B degradation

To investigate the endogenous mechanism maintaining ATP7B homeostasis in PDAC, transcriptomic analyses were first performed and revealed a strong positive correlation between cuproptosis signatures and autophagy activity. ATP7B expression was notably reduced in samples with elevated autophagy (Figure S3A,B), suggesting that autophagy may modulate copper homeostasis and thereby alter cellular susceptibility to cuproptosis. On this basis, ASPC‐1 cells were cultured under serum‐free medium (SFM), low‐glucose (LG) conditions, or EBSS, three classical nutrient‐deprivation regimens that induce graded autophagy activation.

Across all three treatments, ATP7B mRNA expression remained largely unchanged, whereas ATP7B protein levels progressively declined in a time‐dependent manner (Figure S3C **;** Figure 2A), indicating that autophagy primarily controls ATP7B stability post‐transcriptionally. CHX chase assays further substantiated this conclusion, showing that autophagy activation markedly accelerated ATP7B turnover and shortened its half‐life relative to controls (Figure 2B,C). These results demonstrate that ATP7B is selectively removed through autophagy‐mediated protein degradation pathways.

**FIGURE 2:**
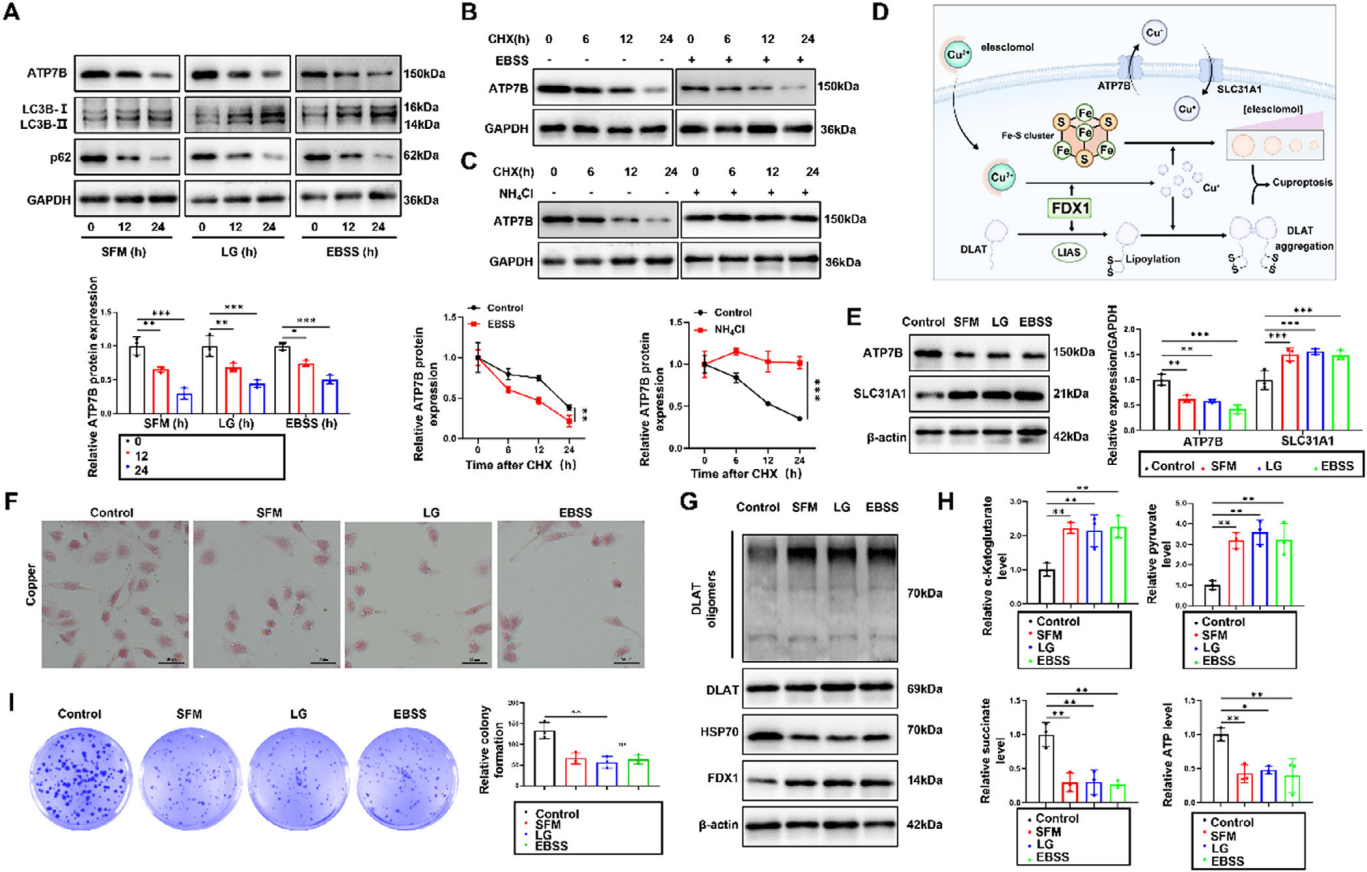
**Autophagy regulates ATP7B stability**. (A) Western blot analysis of ATP7B, LC3, and p62 in ASPC‐1 cells cultured under serum‐free medium (SFM), low‐glucose medium (LG), or EBSS‐induced starvation for the indicated durations (0, 12, and 24 h), with quantitative bar graphs shown at the bottom. (B‐C) Cycloheximide chase assays performed under EBSS treatment or NH_4_Cl inhibition for the indicated durations (0, 6, 12, and 24 h), with corresponding quantification of relative ATP7B protein stability shown at the bottom. (D) Schematic illustration depicting the proposed regulatory model in which elesclomol‐driven copper influx, ATP7B‐mediated copper export, and mitochondrial lipoylated DLAT dynamics collectively modulate cuproptosis signalling. (E) Western blot detection of ATP7B and SLC31A1 expression in cells subjected to SFM, LG, or EBSS treatment for 24 h, with relative quantification shown on the right. (F) Representative copper salt staining images under different culture conditions for 24 h. Scale bar: 50 µm. (G) DLAT oligomerization and protein levels of DLAT, HSP70, and FDX1 in ASPC‐1 cells under the indicated starvation conditions for 24 h. (H) Relative levels of cellular metabolites, including α‐ketoglutarate, pyruvate, succinate, and ATP, in ASPC‐1 cells subjected to control, SFM, LG, or EBSS culture conditions for 24 h. (I) Colony‐forming assays were performed under control, SFM, LG, or EBSS culture conditions for 24 h, followed by standard culture, with representative crystal violet staining and corresponding quantification. Data are presented as mean ± SD. *p < .05, **p < .01, ***p < .001.

SFM, LG, and EBSS treatments led to persistent ATP7B reduction and upregulated SLC31A1 (Figure 2D,E), consequently promoting intracellular Cu^2+^ accumulation and diminishing antioxidant defences, as indicated by reduced GSH and SOD (Figure 2F **;** Figure S2D). Autophagy activation also induced metabolic reprogramming characteristic of cuproptosis, including abnormal accumulation of α‐ketoglutarate, pyruvate, and succinate, together with enhanced DLAT disulfide‐bond aggregation and elevated FDX1 expression (Figure 2G,H **;** Figure S3E). Importantly, in line with intensified cuproptosis, autophagy‐stimulated ASPC‐1 cells displayed markedly impaired colony formation capacity (Figure 2I). Overall, these data demonstrate that autophagy promotes ATP7B degradation and shifts copper homeostasis towards intracellular accumulation, thereby heightening cuproptosis vulnerability in PDAC cells.

### 3.3 Identification of NBR1 as the autophagic cargo receptor responsible for ATP7B turnover

We further excluded proteasomal involvement and found that MG132 treatment did not induce ATP7B accumulation under either dose‐ or time‐escalation condition (Figure S4A), indicating that ATP7B degradation is largely independent of the ubiquitin–proteasome pathway. To identify the autophagy receptor responsible for selective ATP7B clearance, Co‐IP and structural docking analyses were performed, revealing strong interactions with NBR1 and TAX1BP1 (Figure 3A; Figure S4B). However, neither TAX1BP1 knockdown nor disruption of its UBA domain altered ATP7B expression or binding affinity (Figure S4C,D), leading us to focus on NBR1.

**FIGURE 3:**
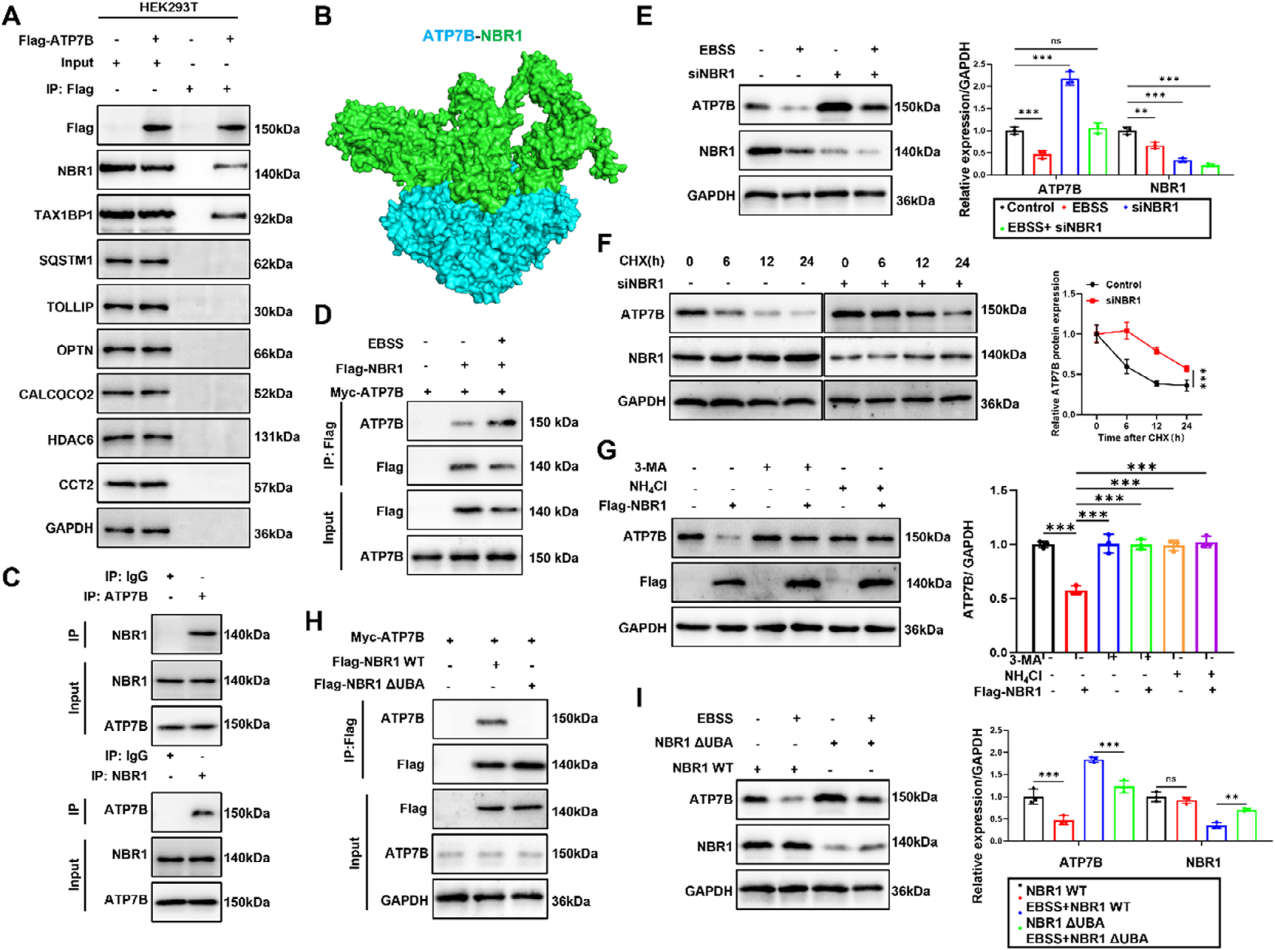
**NBR1 mediates the selective autophagic recognition of ATP7B**. (A) Co‐IP screening in HEK293T cells for ATP7B‐interacting autophagy receptors and cofactors. (B) Structural modelling of the ATP7B–NBR1 complex. (C) Reciprocal Co‐IP validation of the endogenous interaction between ATP7B and NBR1. (D) Co‐IP assay confirming ATP7B–NBR1 interaction under EBSS treatment for 24 h. (E) Protein levels of ATP7B and NBR1 under control or EBSS treatment for 24 h with or without NBR1 knockdown; relative protein quantification shown on the right. (F) CHX chase assay assessing ATP7B protein stability after NBR1 silencing for 0, 6, 12, and 24 h, with relative quantification shown on the right. (G) Immunoblot of ATP7B and Flag‐NBR1 under conditions of autophagy inhibition (3‐methyladenine, 3‐MA) or lysosomal blockade (NH_4_Cl) for 24 h, with relative protein levels quantified on the right. (H) Co‐IP analysis evaluating ATP7B binding to wild‐type NBR1 or UBA‐deleted mutant. (I) Immunoblot detection of ATP7B levels following expression of wild‐type or UBA‐deficient NBR1 under EBSS stimulation for 24 h; corresponding relative protein abundance quantified on the right. Data are presented as mean ± SD. **p < .01, ***p < .001; ns, not significant.

Co‐IP assays confirmed robust ATP7B‐NBR1 interaction, which was further strengthened under EBSS‐induced autophagy activation (Figure 3B–D). NBR1 depletion restored ATP7B protein levels under autophagic stress and delayed its degradation rate in CHX chase assays (Figure 3E,F). Since p62/SQSTM1 and NBR1 often share functional redundancy in selective autophagy, we systematically evaluated their individual contributions to ATP7B degradation. Depletion of NBR1, but not p62, led to a marked accumulation of ATP7B levels, and simultaneous knockdown of both receptors did not yield any additive effect (Figure S4E). These findings suggest that NBR1, rather than p62, serves as the primary cargo receptor mediating ATP7B autophagic degradation. Consistently, inhibition of autophagy at either the early or late stages reversed the ATP7B reduction caused by NBR1 overexpression without affecting its transcription (Figure 3G; Figure S4F). Moreover, loss of the NBR1 UBA domain attenuated ATP7B binding and abolished autophagy‐driven ATP7B degradation (Figure 3H,I). Collectively, these findings identify NBR1 as the selective autophagy receptor for ATP7B, acting through its UBA domain to direct ATP7B towards autophagy‐dependent degradation.

### 3.4 ZBTB10 promotes K63‐linked ubiquitination of ATP7B to enable NBR1‐dependent autophagic degradation

To identify upstream determinants that potentiate ATP7B ubiquitin tagging, particularly the K63‐linked chains required for NBR1 recognition, we first conducted an integrated Venn‐based screening. This strategy pinpointed a single convergent candidate, ZBTB10. In PDAC tissues, both the transcript and protein levels of ZBTB10 were markedly reduced (Figure S5A; Figure S6A–C), and patients with higher ZBTB10 expression exhibited a more favourable prognosis (Figure S5B). Correlation analyses further revealed that ZBTB10 was positively associated with cuproptosis features, while displaying inverse relationships with autophagy activity and ATP7B abundance (Figure S5C–E), suggesting that ZBTB10 may serve as a molecular nexus between ATP7B ubiquitination and cuproptosis sensitivity.

Computational docking and reciprocal co‐immunoprecipitation assays substantiated the physical interaction between ZBTB10 and ATP7B (Figure S6D–F). Consistent with this binding interface, ZBTB10 overexpression prominently enhanced K63‐linked ubiquitination of ATP7B (Figure S6G), indicating its role as a driver of the ubiquitin‐modification pattern. Protein‐turnover analyses further reinforced this mechanism: ZBTB10 depletion attenuated ATP7B degradation and prolonged its half‐life, whereas ZBTB10 overexpression accelerated ATP7B turnover (Figure S6H,I). To further confirm the necessity of ZBTB10 in regulating ATP7B homeostasis, we performed rescue experiments (Figure S7A). Consistently, ZBTB10 depletion resulted in a noticeable accumulation of ATP7B, which was fully reversed upon ZBTB10 rescue (Figure S7B). Moreover, K63‐linked ubiquitination of ATP7B was significantly decreased upon ZBTB10 depletion, but was successfully restored and further enhanced by ZBTB10 re‐expression (Figure S7C).

Functionally, ectopic expression of ZBTB10 consistently reduced ATP7B protein levels across multiple PDAC cell lines (Figure S8A), and the interaction between the two proteins was further strengthened under conditions of autophagy activation (Figure S8B,C). More importantly, ZBTB10 not only increased the abundance of K63‐linked ubiquitin chains on ATP7B, but also augmented the ATP7B–NBR1 interaction and triggered ATP7B degradation. In contrast, autophagy inhibition or NBR1 knockdown mitigated these effects (Figure S8D–F). Notably, this process strictly depended on the UBA domain of NBR1, as UBA‐deficient NBR1 failed to respond to ZBTB10 and could not promote ATP7B degradation (Figure S8G). Moreover, pharmacological blockade of autophagy reversed ZBTB10‐induced ATP7B reduction (Figure S8H), further demonstrating that the regulatory activity of ZBTB10 towards ATP7B homeostasis is contingent upon the autophagy pathway. Taken together, our findings establish that ZBTB10 amplifies K63‐linked ubiquitination of ATP7B, thereby conferring its selective recognition by the UBA domain of NBR1 and facilitating its lysosomal disposal through the autophagy machinery.

### 3.5 ATAUTAC induces selective ATP7B autophagic degradation

Guided by the finding that ATP7B undergoes ZBTB10‐mediated K63 ubiquitination and NBR1‐dependent autophagic clearance, we developed an ATAUTAC (Figure 4A). ATAUTAC did not alter ATP7B transcripts but induced a time‐dependent protein reduction (Figure 4B–D **;** Figure S9A) while enhancing K63‐linked ubiquitination (Figure 4E,F). Furthermore, the lysosomal dependency of this clearance was confirmed, as the lysosomal inhibitor chloroquine rescued ATP7B from ATAUTAC‐mediated degradation (Figure 4G). Cellular thermal shift assay (CETSA) demonstrated direct binding of ATAUTAC to ATP7B and a marked reduction in ATP7B thermal stability upon compound engagement (Figure 4H). Importantly, ATAUTAC strengthened ATP7B–NBR1 association and accelerated ATP7B degradation, both of which were abolished upon NBR1 suppression (Figure 4I–K). To verify the selectivity of ATAUTAC, we monitored the expression of related copper transporters and chaperones. The abundance of ATP7A, Antioxidant 1 copper chaperone (ATOX1), and copper chaperone for superoxide dismutase (CCS) remained unchanged upon compound treatment, whereas SLC31A1 showed a compensatory increase, likely adapting to export blockade (Figure S9B,C). These data demonstrate that ATAUTAC promotes selective ATP7B elimination by amplifying its K63 ubiquitination and enforcing NBR1‐mediated autophagic degradation.

**FIGURE 4:**
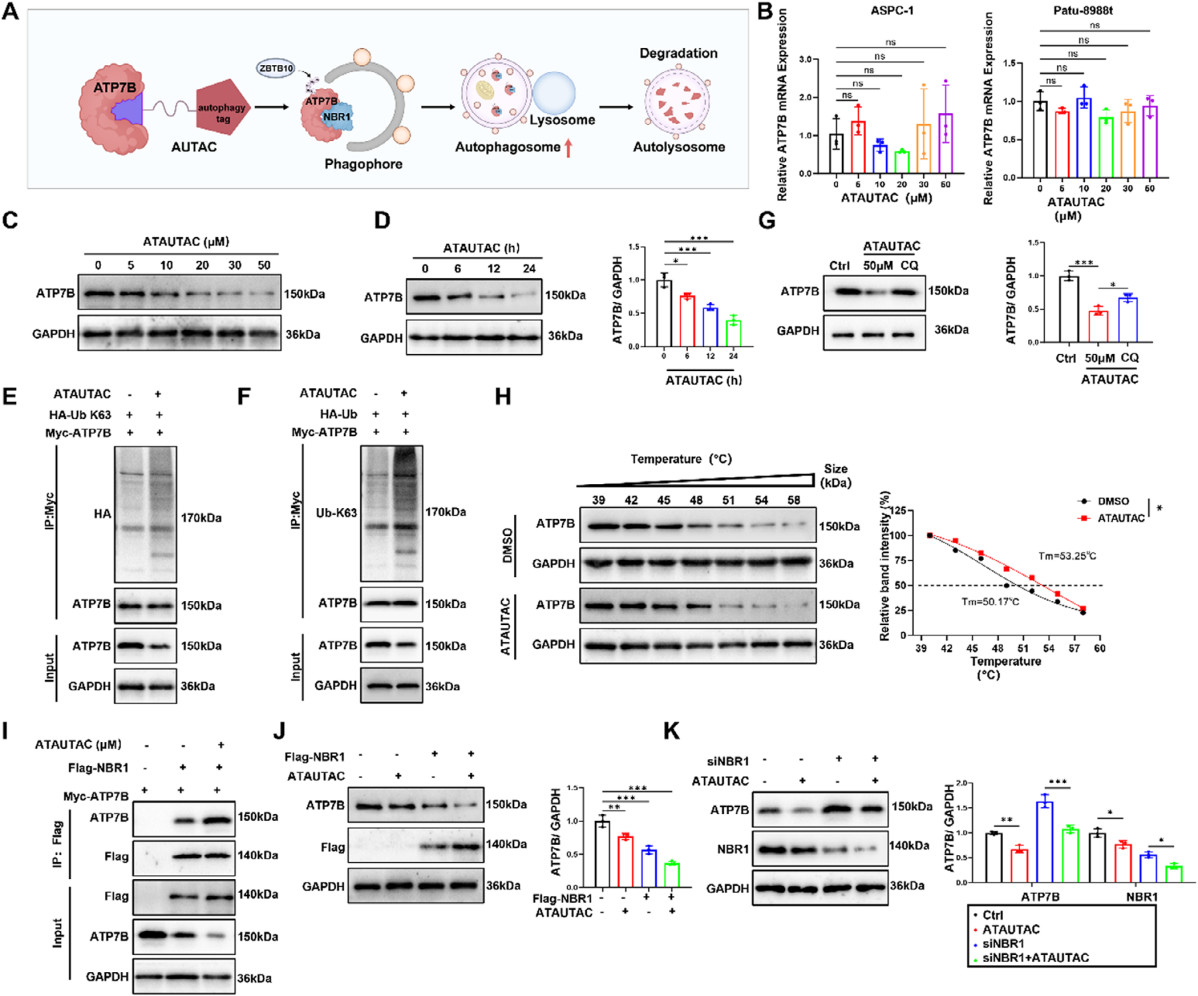
**ATAUTAC induces autophagic targeting of ATP7B**. (A) Schematic illustration of the ATAUTAC‐induced autophagic degradation pathway targeting ATP7B, involving ZBTB10‐mediated ubiquitin tagging, NBR1 recognition, and autolysosomal degradation. (B) qPCR analysis of ATP7B mRNA levels in ASPC‐1 and Patu‐8988t cells treated with the indicated concentrations of ATAUTAC (0, 5, 10, 20, 30, and 50 µM) for 24 h. (C) Immunoblot analysis of ATP7B protein expression following ATAUTAC treatment at the indicated doses (0, 5, 10, 20, 30, and 50 µM) for 24 h. (D) Immunoblot detection of ATP7B protein levels in ASPC‐1 cells exposed to ATAUTAC (50 µM) for the indicated time periods (0, 6, 12, and 24 h), accompanied by a bar graph for relative quantification. (E‐F) Co‐IP of Myc‐ATP7B demonstrating K63‐linked ubiquitination in the presence of ATAUTAC (50 µM) for 24 h, assessed using HA‐Ub‐K63 or HA‐Ub constructs and immunoblotting with the indicated antibodies. (G) Protein levels of ATP7B in ASPC‐1 cells treated with control, ATAUTAC (50 µM), or lysosomal inhibitor chloroquine (CQ, 50 µM) for 24 h, with relative quantification shown on the right. (H) Cellular thermal shift assay (CETSA) showing ATP7B protein stability in ASPC‐1 cells treated with ATAUTAC (50 µM) or DMSO across the indicated temperature gradients, with densitometry‐based melting curves (n = 3 independent biological replicates). (I) Co‐IP analysis of the interaction between NBR1 and ATP7B under ATAUTAC (50 µM) treatment for 24 h, followed by immunoblot detection. (I) Immunoblot analysis of ATP7B expression following NBR1‐WT overexpression with or without ATAUTAC (50 µM) for 24 h, with relative protein levels quantified by bar graphs. (J) Immunoblot assessment of ATP7B levels in cells transfected with or without siNBR1 and treated with ATAUTAC (50 µM) for 24 h, with a corresponding bar graph showing relative protein levels. Data are presented as mean ± SD. *p < .05, **p < .01, ***p < .001; ns, not significant.

### 3.6 ATAUTAC enhances copper stress and cuproptosis following ATP7B depletion

Based on ATAUTAC‐driven ATP7B depletion, we further examined whether ATAUTAC promotes cuproptosis in PDAC cells. ATAUTAC treatment triggered progressive metabolic perturbations in ASPC‐1 cells and the mouse pancreatic cancer cell line Panc02, reflected by increased α‐ketoglutarate and pyruvate alongside reduced succinate and ATP (Figure S10A; Figure S11A). This metabolic shift paralleled increased intracellular Cu^2^^+^ and attenuated antioxidant capacity (Figure S10B;C; Figure S11B,C). Correspondingly, cuproptosis signatures, including FDX1 and lipoylated DLAT/DLST, were reinforced while ATP7B declined (Figure S10D–F; Figure S11D–F), and mitochondrial ROS accumulation intensified (Figure S10G,H **;** Figure S11G,H). Functionally, ATAUTAC curtailed proliferation and colony formation and reduced cell viability in a dose‐dependent manner (Figure S10I–K **;** Figure S11I,J).

To confirm that ATAUTAC‐induced cell death is indeed driven by cuproptosis rather than other modalities, we performed pharmacological rescue assays. Cell viability was successfully restored only by the copper chelator tetrathiomolybdate (TTM), whereas inhibitors of pyroptosis (VX‐765), apoptosis (Z‐VAD‐FMK), necroptosis (Necrostatin‐1), and ferroptosis (Fer‐1) failed to prevent cell death (Figure 5A). Consistently, TTM treatment effectively rescued the colony‐forming capacity of ASPC‐1 and Patu‐8988t cells (Figure 5B) and significantly limited intracellular copper salt accumulation (Figure 5C) under ATAUTAC exposure. Furthermore, to confirm the genetic dependency of this process on the cuproptosis pathway, we silenced the key regulator FDX1. FDX1 depletion significantly alleviated ATAUTAC‐induced metabolic perturbations, partially restoring ATP and succinate levels while reducing α‐ketoglutarate and pyruvate (Figure 5D). This was accompanied by a noticeable decrease in intracellular copper concentration (Figure 5E), restoration of GSH and SOD levels (Figure 5F), and reduced copper salt accumulation (Figure 5G). At the protein level, FDX1 knockdown mitigated the upregulation of lipoylated DLAT/DLST and SLC31A1 (Figure 5H,I). Consistently, FDX1 silencing significantly silenced mitochondrial ROS accumulation (Figure 5J) and successfully rescued both colony‐forming capacity (Figure 5K) and overall cell viability (Figure 5L) under ATAUTAC treatment. Together, these results demonstrate that ATAUTAC selectively triggers cuproptosis and copper toxicity following ATP7B depletion.

**FIGURE 5:**
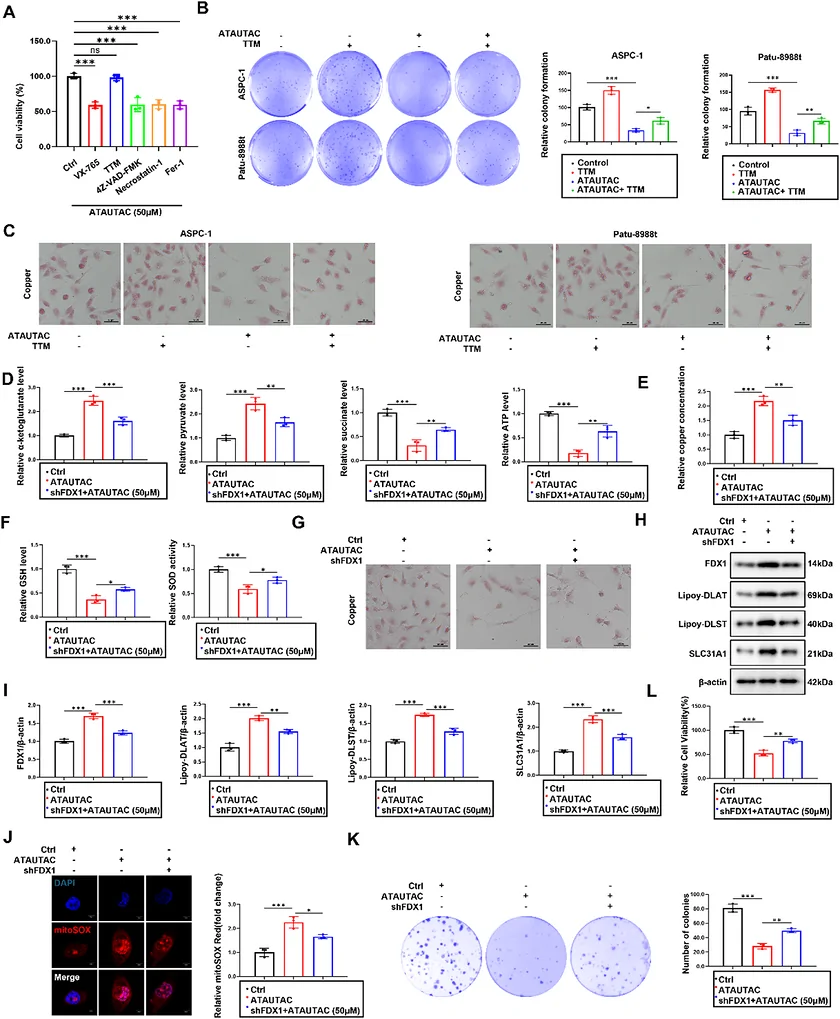
**ATAUTAC triggers canonical cuproptosis following ATP7B depletion**. (A) Relative cell viability of Patu‐8988t cells treated with ATAUTAC (50 µM) with or without standard pathway inhibitors, including VX‐765 (10 µM), TTM (50 µM), Z‐VAD‐FMK (20 M), Necrostatin‐1 (10 µM), or Ferrostatin‐1 (2 µM) for 24 h. (B) Colony‐forming assays of ASPC‐1 and Patu‐8988t cells treated with control, TTM (50 µM), ATAUTAC (50 µM), or combined ATAUTAC+TTM for 24 h, with corresponding quantification shown on the right. (C) Representative copper salt staining images in ASPC‐1 and Patu‐8988t cells under the indicated treatment conditions for 24 h. Scale bar: 50 µm. (D) Relative levels of cellular metabolites, including α‐ketoglutarate, pyruvate, succinate, and ATP, in ASPC‐1 cells under control, ATAUTAC (50 µM), or shFDX1+ATAUTAC (50 µM) treatment for 24 h. (E) Relative copper concentration in ASPC‐1 cells in the indicated treatment groups for 24 h. (F) Relative levels of intracellular GSH and SOD activity in ASPC‐1 cells in the indicated treatment groups for 24 h. (G) Representative copper salt staining images in ASPC‐1 cells under control, ATAUTAC (50 µM), or shFDX1+ATAUTAC (50 µM) conditions for 24 h. Scale bar: 50 µm. (H, I) Protein levels of FDX1, lipoylated DLAT, lipoylated DLST, and SLC31A1 in ASPC‐1 cells in the indicated treatment groups for 24 h, with corresponding quantitative bar graphs shown in (I). (J) Mitochondrial ROS accumulation (MitoSOX) in ASPC‐1 cells under control, ATAUTAC (50 µM), or shFDX1+ATAUTAC (50 µM) treatment for 24 h, with relative quantification shown on the right. (K) Colony‐forming assays of ASPC‐1 cells under control, ATAUTAC (50 µM), or shFDX1+ATAUTAC (50 µM) treatment for 24 h, followed by standard culture, with corresponding quantification shown on the right. (L) Relative cell viability of ASPC‐1 cells in the indicated treatment groups for 24 h. Data are presented as mean ± SD. *p < .05, **p < .01, ***p < .001; ns, not significant.

### 3.7 ATAUTAC suppresses PDAC growth in vivo

To assess the in vivo therapeutic potential of ATAUTAC, ASPC‐1 xenograft–bearing mice were treated with ATAUTAC (20 or 40 mg/kg). ATAUTAC administration led to a clear suppression of tumour progression, evidenced by substantial reductions in tumour volume and weight (Figure 6A–C). Notably, the treatment exhibited favourable tolerability, as neither body‐weight loss nor pathological abnormalities in major organs were detected, even at 40 mg/kg (Figure S12A–C).

**FIGURE 6:**
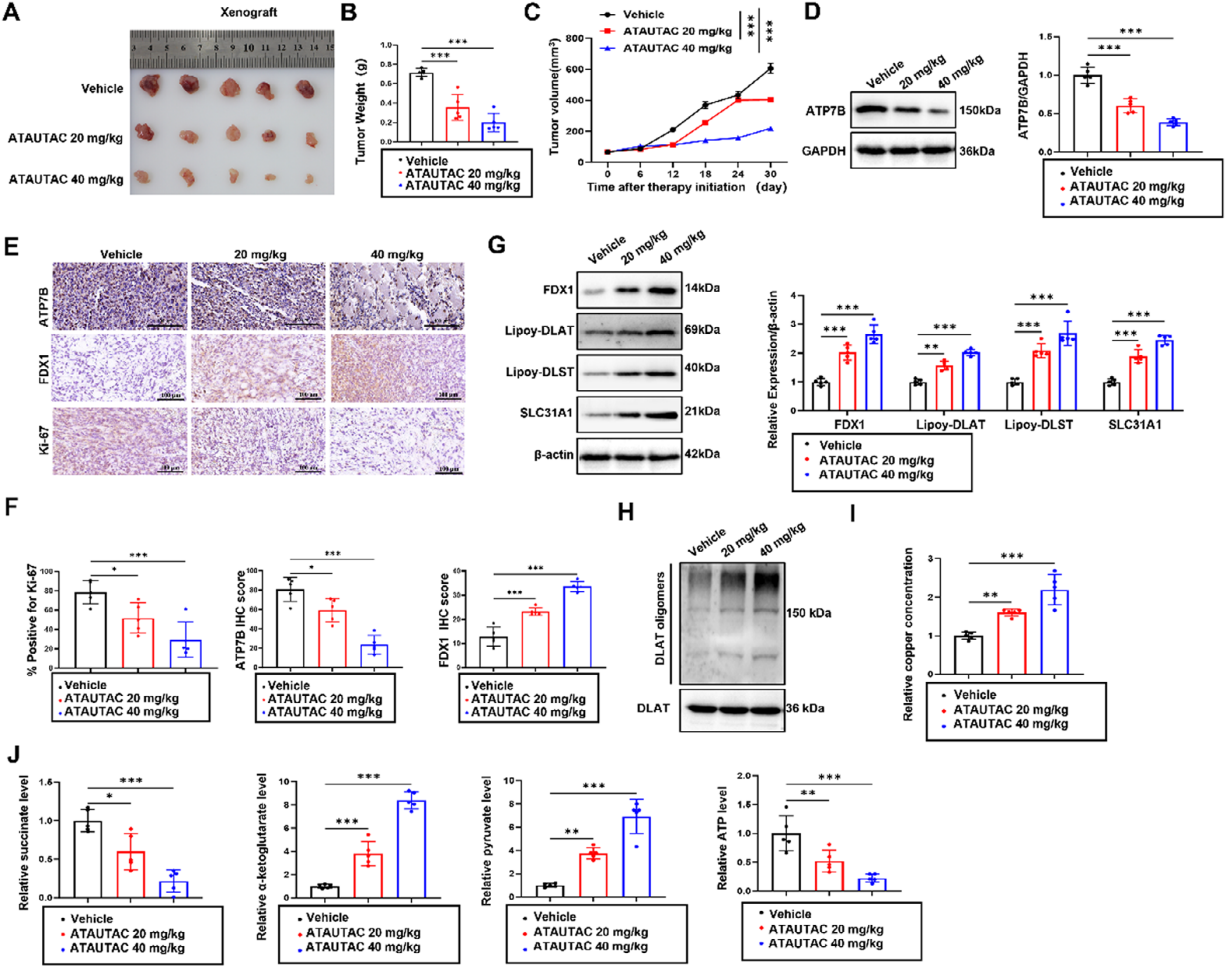
**In vivo evaluation of ATAUTAC in PDAC xenograft models**. (A) Gross images of ASPC‐1 xenograft tumours in female BALB/c nude mice treated intratumorally with vehicle, 20 mg/kg, or 40 mg/kg ATAUTAC every 2–3 days for 30 days (*n* = 5 mice per group). (B) Quantification of excised tumour weight in each treatment group at the experimental endpoint (*n* = 5 mice per group). (C) Tumour growth curves of xenografts monitored over the 30‐day treatment period. (D) Protein levels of ATP7B in tumour lysates from the indicated treatment groups, with relative protein levels quantified by bar graphs. (E) Representative immunohistochemical staining of ATP7B, FDX1, and Ki‐67 in xenograft sections. Scale bar: 100 µm. (F) Quantitative analysis of immunohistochemical scores for Ki‐67, ATP7B, and FDX1 in the indicated groups. (G) Protein levels of FDX1, lipoylated DLAT, lipoylated DLST, and SLC31A1 in tumour lysates, with relative protein levels represented by bar graphs. (H) DLAT oligomerization in tumour lysates analyzed under nonreducing conditions. (I) Relative intratumoral copper concentration in xenograft tissues from the indicated treatment groups. (J) Relative levels of succinate, α‐ketoglutarate, pyruvate, and ATP in tumour lysates from the indicated treatment groups. Data are presented as mean ± SD. *p < .05, **p < .01, ***p < .001; ns, not significant.

Mechanistically, ATAUTAC treatment led to a pronounced suppression of ATP7B in tumour tissues and a concomitant reinforcement of cuproptosis markers, including FDX1 and lipoylated DLAT/DLST (Figure 6D–H). These molecular changes coincided with metabolic features of copper stress, characterized by lowered ATP and succinate and elevated intracellular Cu^2+^, α‐ketoglutarate, and pyruvate (Figure 6I,J). Together, the in vivo findings demonstrate that ATAUTAC effectively downregulates ATP7B, amplifies metabolic signatures of copper toxicity, and impedes PDAC tumour progression with favourable tolerability, supporting its translational potential as a cuproptosis‐sensitizing strategy.

### 3.8 ATAUTAC potentiates GEM efficacy by aggravating copper stress in PDAC

Given that acquired resistance limits the clinical benefit of GEM in PDAC, we next explored whether ATAUTAC could reinforce GEM sensitivity. In vitro, dual treatment with ATAUTAC and GEM synergistically impaired the proliferative capacity of ASPC‐1 and Patu‐8988t cells and induced a more pronounced intracellular Cu^2^^+^ accumulation than either agent alone (Figure S13), suggesting that ATAUTAC‐elicited copper stress may cooperate with GEM cytotoxicity.

Consistent with these observations, PDAC xenografts receiving the combination regimen according to the therapeutic schedule (Figure 7A) showed significantly prolonged survival compared with the other treatment groups (Figure 7B). Consistently, the combination therapy exhibited the most profound tumour growth inhibition, as evidenced by a substantial reduction in both excised tumour weight (Figure 7C,D) and tumour volume (Figure 7E). At the molecular level, this growth suppression was accompanied by reinforced cuproptosis and copper‐import markers in tumour tissues (Figure 7F,G). Histological profiling confirmed that the combination therapy suppressed proliferative activity, depleted ATP7B, and elevated FDX1 levels, yielding the most prominent alterations compared with either monotherapy (Figure 7H,I).

**FIGURE 7:**
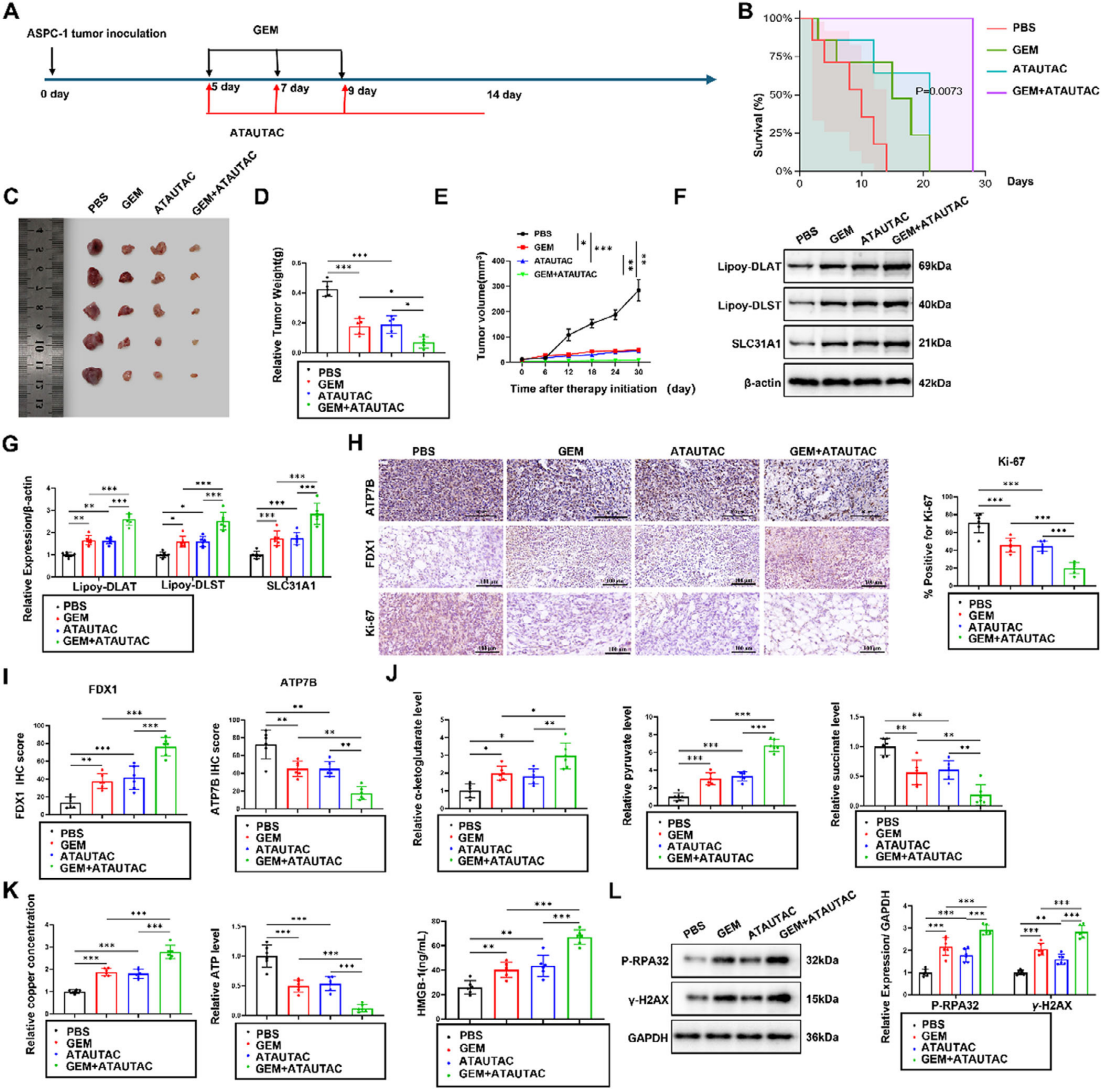
**ATAUTAC enhances GEM therapeutic response in PDAC xenografts**. (A) Schematic representation of therapeutic schedule for ASPC‐1 xenograft‐bearing mice treated with gemcitabine (GEM, 40 mg/kg dissolved in saline) and/or ATAUTAC (40 mg/kg dissolved in a vehicle of 10% DMSO, 40% PEG300, 5% Tween‐80, and 45% saline) on days 5, 7, and 9, and thereafter every 2 days for 14 days (*n* = 5 mice per group). (B) Kaplan–Meier survival curves of mice under the indicated treatments (*n* = 5 mice per group). (C) Representative images of xenograft tumours excised at the endpoint (*n* = 5 mice per group). (D) Quantification of tumour weight from each treatment group (*n* = 5 mice per group). (E) Tumour growth curves showing volume measurements recorded throughout the treatment course (*n* = 5 mice per group). (F) Protein levels of lipoylated DLAT, lipoylated DLST, and SLC31A1 in xenograft tissues (F), with corresponding relative protein expression quantified by bar graphs shown in (G). (H) Representative immunohistochemical staining of ATP7B, FDX1, and Ki‐67 in tumour sections from each group, with relative quantification of Ki‐67‐positive cells shown on the right. Scale bar: 100 µm. (I) Quantitative scoring of immunohistochemical staining for FDX1 and ATP7B in tumour sections. (J) Relative levels of metabolites associated with TCA flux, including α‐ketoglutarate, pyruvate, and succinate, in xenograft tumour lysates. (K) Relative levels of intratumoral Cu^2+^ concentration, relative ATP levels, and absolute concentration of HMGB1 (ng/mL) in xenograft tumours from the indicated groups. (L) Protein levels of replication stress marker P‐RPA32 and DNA damage marker γ‐H2AX in tumour lysates, with corresponding relative protein expression represented by bar graphs. Data are presented as mean ± SD. *p < .05, **p < .01, ***p < .001.

Metabolically, combined treatment further aggravated copper‐dependent mitochondrial dysfunction, evidenced by elevated α‐ketoglutarate and pyruvate levels alongside reduced succinate (Figure 7J). These shifts paralleled increased intratumoral copper concentration, depleted ATP levels, and elevated levels of the immunogenic cell death marker HMGB1 (Figure 7K). To explore the molecular mechanism underlying this synergy, we evaluated markers of replication stress and DNA damage in xenograft tissues. While GEM monotherapy induced moderate replication stress, the combination of ATAUTAC and GEM synergistically and robustly elevated the levels of both phosphorylated RPA32 and the DNA double‐strand break marker γ‐H2AX (Figure 7L). This indicates that the cuproptosis‐driven metabolic perturbations induced by ATAUTAC severely compromise the cells’ metabolic capacity to handle the genotoxic stress imposed by GEM, thereby exacerbating replication stress and DNA damage.

Importantly, neither ATAUTAC monotherapy nor its use in combination with GEM induced overt systemic toxicity, as reflected by stable body weight, unchanged organ coefficients, and normal serum biochemical indices (Figure S14). Collectively, these findings indicate that ATAUTAC augments the therapeutic efficacy of GEM by amplifying copper stress and cuproptosis susceptibility, offering a mechanistically grounded strategy to overcome chemoresistance in PDAC.

## 4 DISCUSSION

PDAC remains a therapeutically refractory malignancy owing to its profound metabolic adaptability and chemoresistance. Delineating these metabolic defence mechanisms is critical to identifying druggable vulnerabilities. Here, we established ATP7B as a key determinant of copper homeostasis in PDAC, whose aberrant elevation correlates with poor prognosis. Genetic suppression of ATP7B reinstates copper‐dependent metabolic stress and restrains tumour growth, exposing an inherent liability to cuproptosis. Building on this, we developed ATAUTAC, an autophagy‐dependent degrader that selectively eliminates ATP7B to amplify copper‐driven mitochondrial metabolic perturbations and augment GEM efficacy, offering a mechanistically grounded sensitization strategy.

Cuproptosis is mechanistically centred on the TCA cycle,^22^ where copper directly binds lipid‐acylated DLAT, leading to abnormal protein aggregation and depletion of Fe–S cluster proteins. Our study demonstrates that PDAC cells exploit ATP7B overexpression to facilitate copper efflux, thereby sustaining mitochondrial functionality and evading cuproptosis under metabolically demanding conditions. While ATP7B is traditionally implicated in cisplatin extrusion^23^, ^24^ and chemotherapy resistance,^25^, ^26^ its functional involvement in cuproptosis regulation in PDAC has remained uncharacterized. Here, we establish this link and provide molecular evidence that ATP7B‐governed copper homeostasis constitutes a crucial survival advantage in PDAC.

Importantly, our study delineates a previously unrecognized role of autophagy in orchestrating ATP7B protein homeostasis. We observed that autophagy activation selectively reduces ATP7B protein abundance post‐translationally,^27^, ^28^ concurrently triggering cuproptosis pathway activation. These findings indicate that ATP7B removal per se is sufficient to remodel cellular copper susceptibility and re‐engage cuproptosis stress, consistent with emerging evidence linking autophagic processes to cuproptosis sensitivity.^29^, ^30^, ^31^

Mechanistically, we identified NBR1 as the selective autophagy receptor mediating ATP7B clearance via its UBA domain. Although NBR1 is structurally homologous to p62 and is recognized as a receptor for K63‐linked ubiquitinated substrates,^32^, ^33^ its role in regulating copper transport and metabolic resistance has not been reported. We demonstrated that ATP7B undergoes ZBTB10‐driven K63‐linked ubiquitination—rather than the K48‐type associated with proteasomal degradation—and is subsequently recognized and directed to autophagic degradation by NBR1. This aligns with the consensus that K63 chains serve as privileged signals for autophagic disposal.^34^, ^35^ Crucially, ZBTB10 acts as the upstream driver that sculpts this ubiquitin landscape. While ZBTB family members are traditionally considered transcriptional regulators,^36^, ^37^, ^38^ our work reveals ZBTB10 as a functional orchestrator of K63 linkage assembly and autophagy initiation, expanding its biological scope into metabolic governance in cancer.

Leveraging these mechanistic insights, we developed ATAUTAC, the first AUTAC molecule targeting ATP7B. By binding ATP7B to enhance K63 ubiquitination and prompt NBR1‐mediated selective autophagy, ATAUTAC dismantles the copper‐buffering barrier at its origin with favourable in vivo tolerability. This “removal of protective buffering” rather than a “forced cytotoxic insult” underscores a highly precise and safety‐oriented approach to exploiting metabolic vulnerabilities in PDAC.

Finally, our study proposes a translational combination strategy in which ATAUTAC synergizes with GEM. While ATP7B establishes a crucial copper homeostatic shield for tumour survival, GEM resistance is often driven by nucleotide metabolic reprogramming and enhanced DNA damage repair.^39^, ^40^ By weakening the copper efflux barrier, ATAUTAC re‐sensitizes PDAC cells to the metabolic and genotoxic stress imposed by GEM, augmenting cell death and improving therapeutic efficacy in vivo. These findings establish cuproptosis sensitization as a viable conceptual framework to overcome chemotherapy resistance via rational combinatorial regimens.

Despite these promising findings, several translational challenges and limitations of our study must be acknowledged. First, ATAUTAC was administered via intratumoral injection in our in vivo models, which is not a standard route of clinical administration for PDAC. To achieve broader clinical applicability, future studies are warranted to optimize the systemic pharmacokinetics and bioavailability of ATAUTAC. In this regard, encapsulating ATAUTAC in targeted nano‐delivery systems, such as tumour‐specific nanoparticles or liposomes, represents a promising strategy to facilitate systemic delivery and maximize tumour accumulation. Second, although our subcutaneous xenograft models demonstrated the therapeutic potential of ATAUTAC, they do not fully recapitulate the dense, desmoplastic stroma and complex microenvironment characteristic of human PDAC. Validating this therapeutic approach in orthotopic pancreatic transplantation models or genetically engineered mouse models (such as the KPC model) is an essential next step to provide more clinically relevant insights into drug distribution, stroma‐penetrating capability, and therapeutic efficacy.

## 5 CONCLUSION

In summary, this work identifies ATP7B as a key molecular barrier to cuproptosis in PDAC and demonstrates that its selective removal via the autophagy‐based degrader ATAUTAC constitutes an effective, pharmacologically tractable strategy. By disrupting intracellular copper homeostasis, ATAUTAC amplifies metabolic vulnerability and restrains tumour progression, establishing cuproptosis sensitization as a viable therapeutic concept. These findings highlight ATAUTAC‐mediated ATP7B degradation as a promising avenue for PDAC treatment, and underscore the broader potential of targeting copper homeostasis in solid tumours, either as monotherapy or in rational combination regimens.

## AUTHOR CONTRIBUTIONS

Lei Zhang and Jian‐ang Li: responsible for research design, data auditing, and manuscript review. Zihan Zhang, Min Li, and Jiale Feng: Conducted experiments, performed data analysis, and drafted the manuscript. Lei Zhang serves as the principal corresponding author and takes the primary responsibility for communication with the journal and coordination of the publication process. All authors declare that they have made substantial contributions to the conception or design of the work; or the acquisition, analysis, or interpretation of data for the work; and have participated in drafting the work or revising it critically for important intellectual content; and have given final approval of the version to be published; and agree to be accountable for all aspects of the work in ensuring that questions related to the accuracy or integrity of any part of the work are appropriately investigated and resolved.

## CONFLICT OF INTEREST STATEMENT

The authors declare no conflicts of interest.

## FUNDING INFORMATION

The authors have nothing to report.

## ETHICS STATEMENT

This study was approved by the Ethical Approval for Research Involving Animals of Zhongshan Hospital, Fudan University (2025‐02‐19).

## CONSENT TO PARTICIPATE

The authors have nothing to report.

## CONSENT FOR PUBLICATION

The authors have nothing to report.

## Supporting information



Supporting Information

Supporting Information

## Data Availability

The data analyzed during the current study are available from the corresponding author on reasonable request.
