# Supplementary material for: Selective AUTAC‐mediated ATP7B degradation unlocks cuproptosis vulnerability in pancreatic ductal adenocarcinoma
Source: Clin Transl Med. 2026 Oct 27;16(11):e70848. doi: 10.1002/ctm2.70848 (PMC13714097; doi:10.1002/ctm2.70848)
Supplement: Supplementary file 1 [file CTM2-16-e70848-s001.docx]

**Supplementary Methods and Materials**

1. **Colony Formation Assay**

Cells (500–1,000/well) were seeded in 6-well plates and cultured for 10–14 days. Formed colonies were fixed with 4% paraformaldehyde, stained with 0.1% crystal violet, and counted (≥50-cell definition) using ImageJ.

2. **Western Blot Analysis**

Cells or tumor tissues were lysed in RIPA buffer supplemented with protease and phosphatase inhibitors. Total protein concentration was measured using a BCA assay. Equal amounts of protein were separated by SDS–PAGE and transferred onto PVDF membranes. After blocking with 5% non-fat milk or BSA for 1 h at room temperature, membranes were incubated with primary antibodies at 4 °C overnight, followed by HRP-conjugated secondary antibodies for 1 h at room temperature. Protein signals were detected using enhanced chemiluminescence reagents and captured with a ChemiDoc imaging system. Band intensities were quantified with ImageJ software and normalized to internal loading controls.

3. **Antibodies**

ATP7B (19786-1-AP, Proteintech), FDX1 (ab108257, Abcam), SLC31A1 (27499-1-AP, Proteintech), LC3B-Ⅰ/Ⅱ (18725-1-AP, Proteintech), p62 (31403-1-AP, Proteintech), DLAT (13426-1-AP, Proteintech), Lipoy-DLAT (13426-1-AP, Proteintech), Lipoy-DLST (31174-1-AP, Proteintech), HSP70 (10995-1-AP, Proteintech), NBR1 (16004-1-AP, Proteintech), TAX1BP1 (#5105, CellSignalingTechnology), TOLLIP (11315-1-AP, Proteintech), OPTN (10837-1-AP, Proteintech), CALCOCO2 (12229-1-AP, Proteintech), HDAC6 (ab117516, Abcam), CCT2 (24896-1-AP, Proteintech), ZBTB10 (ab117786, Abcam), Ki-67 (27309-1-AP, Proteintech), GAPDH (60004-1-Ig, Proteintech), β-actin (66009-1-Ig, Proteintech).

4. **Immunohistochemistry (IHC)**

Paraffin-embedded tumor tissues were sectioned at 4 µm, deparaffinized in xylene, and rehydrated through graded ethanol. Antigen retrieval was performed in citrate buffer (pH 6.0) using a pressure cooker. Endogenous peroxidase activity was blocked with 3% hydrogen peroxide, followed by incubation with 5% BSA to block nonspecific binding. Sections were then incubated with primary antibodies at 4 °C overnight, washed, and incubated with HRP-conjugated secondary antibodies for 1 h at room temperature. Signal development was performed using DAB substrate, and nuclei were counterstained with hematoxylin. Slides were dehydrated, mounted, and imaged using a light microscope. The staining intensity and positive area were quantified by ImageJ software.

5. **Ubiquitination Assay**

Cells were lysed in IP lysis buffer (Beyotime) supplemented with protease inhibitors and 10 mM MG132 to prevent ubiquitin degradation. Equal amounts of protein were incubated with the indicated primary antibodies overnight at 4 °C, followed by incubation with protein A/G magnetic beads for 2–4 h at 4 °C. Immunocomplexes were washed extensively, eluted by boiling in SDS loading buffer, and analyzed by immunoblotting with ubiquitin-specific antibodies. For selective detection of K63-linked ubiquitination, membranes were probed with anti-K63-Ub antibodies. When required, HA-tagged ubiquitin plasmids were transfected prior to MG132 treatment to enhance ubiquitin signal.

6. **Co-immunoprecipitation (Co-IP)**

Cells were lysed in lysis buffer (IP buffer) containing protease and phosphatase inhibitors. The protein concentration was determined using the BCA assay. Equal amounts of lysate were incubated overnight with the indicated primary antibodies (1–2 µg) at 4°C. Protein A/G magnetic beads (10 µL) were added and incubated for an additional 2–4 hours at 4°C. The beads were washed extensively with lysis buffer to remove non-specifically bound proteins. The immunocomplexes were eluted in SDS loading buffer and analyzed by SDS-PAGE and immunoblotting using specific secondary antibodies.

7. **Cellular Thermal Shift Assay (CETSA)**

Cells were harvested and resuspended in PBS at a concentration of 1 × 10^7 cells/mL. The cell suspension was treated with the indicated compound(s) for 1 hour at room temperature, followed by incubation at various temperatures (range: 37°C to 60°C) for 3 minutes. Cells were then cooled on ice for 5 minutes and centrifuged at 10,000 × g for 5 minutes to separate the soluble fraction from the heat-denatured fraction. The supernatant and pellet were collected and analyzed by SDS-PAGE and immunoblotting. Protein thermal stability was assessed by comparing the amount of target protein in the soluble and insoluble fractions across different temperatures.

8. **Measurement of Metabolite Levels**

The levels of pyruvate (S0299S, Beyotime), succinate (S0529S, Beyotime), α-KG (S0323S, Beyotime), ATP (S0026, Beyotime), GSH (S0052, Beyotime), and SOD (S0101S, Beyotime) were determined using commercially available assay kits according to the manufacturer’s protocols. To eliminate baseline variations in cell number and density, the absolute raw values of these metabolites were normalized to the total protein concentration of each respective sample, and the final data were presented as relative levels.

9. **Quantitative PCR (qPCR)**

Total RNA was extracted from cells or tissues using the TRIzol reagent (Invitrogen) according to the manufacturer's instructions. RNA concentration and quality were assessed using a Nanodrop spectrophotometer. For cDNA synthesis, 1 μg of RNA was reverse transcribed using the PrimeScript RT Reagent Kit (Takara) according to the manufacturer's protocol. qPCR amplification was performed using the SYBR Green PCR Master Mix (Applied Biosystems) on an Applied Biosystems 7500 Real-Time PCR System. The reaction conditions were as follows: initial denaturation at 95°C for 10 minutes, followed by 40 cycles of denaturation at 95°C for 15 seconds, and annealing/extension at 60°C for 1 minute. The relative expression levels of ATP7B were normalized to the housekeeping gene GAPDH or β-actin, and calculated using the 2^-ΔΔCt method. The following primers were used for ATP7B and GAPDH: ATP7B forward: 5’-GGACCACAACATCATTCCAGGAC-3’, ATP7B reverse: 5’-ATGAGCACGTCCATGTGTGTGTGTG-3’, GAPDH forward: 5’-GTCTCCTCTCTGACTTCAACAGCG-3’, GAPDH reverse: 5’-ACCACCCTGTGTGTGTGTGTGTGTAGCCAA-3’.

**10. HMGB1 ELISA**

Extracellular and intratumoral HMGB1 concentrations were determined using a commercially available Human HMGB1 ELISA Kit (E-EL-H1554, Elabscience, Wuhan, China) according to the manufacturer’s instructions. Briefly, cell culture supernatants or tumor tissue homogenate lysates were added to antibody-precoated microplate wells. After incubation and washing steps, horseradish peroxidase (HRP)-conjugated antibody was introduced, followed by substrate solution. The absorbance was measured at 450 nm using a microplate reader.

**11. Design, synthesis and characterization of ATP7B-targeting ATAUTAC**

ATAUTAC was designed as a bifunctional autophagy-targeting chimera consisting of an ATP7B-recognition module, a flexible PEG linker, and an autophagy-recruiting module. The overall design was based on a modular AUTAC construction strategy, in which a target-binding ligand and an autophagy-recruiting tag are separately prepared and subsequently conjugated through a linker to generate a bifunctional degrader. Briefly, the ATP7B-recognition module was selected to confer target specificity toward ATP7B, whereas the autophagy-recruiting module was introduced to promote selective autophagic degradation of the target protein. The two modules were connected through a PEG-based linker to provide sufficient spatial flexibility for simultaneous ATP7B engagement and autophagy recruitment. The ATP7B-targeting ligand and the autophagy-recruiting tag were chemically modified to introduce complementary alkyne and azide groups, respectively. The final ATAUTAC molecule was then obtained by Cu(I)-catalyzed azide–alkyne cycloaddition.

In brief, the azide-containing autophagy-recruiting intermediate and the alkyne-containing ATP7B-recognition intermediate were dissolved in anhydrous DMF, followed by the addition of sodium ascorbate and CuSO_4_·5H_2_O under an ice-water bath. The reaction mixture was stirred at 0 ℃ for 24 h, quenched with water, extracted with dichloromethane, dried over anhydrous MgSO_4_, filtered, and concentrated under reduced pressure. The resulting product was purified according to standard chromatographic procedures. For *in vitro* assays, ATAUTAC was dissolved in DMSO to prepare stock solutions and stored at -80 ℃ protected from light. Before treatment, the stock solution was freshly diluted in culture medium to the indicated working concentrations, and the final DMSO concentration was kept below 0.1% in all groups.
