# Supplementary material for: Selective AUTAC‐mediated ATP7B degradation unlocks cuproptosis vulnerability in pancreatic ductal adenocarcinoma
Source: Clin Transl Med. 2026 Oct 27;16(11):e70848. doi: 10.1002/ctm2.70848 (PMC13714097; doi:10.1002/ctm2.70848)
Supplement: Supplementary file 2 [file CTM2-16-e70848-s002.docx]

**Supplementary Figures**

**Supplementary Figure S1. Clinical relevance and physiological feedback regulation of ATP7B in PDAC.**

(A) Kaplan–Meier survival curves of PDAC patients in the GSE57495 dataset grouped by ATP7B expression levels.

(B) Relative mRNA levels of ATP7B in paired adjacent normal (N) and tumor (T) pancreatic tissues from PDAC patients.

(C) Relative expression levels of ATP7B across pathological stages (Stage I, II, III, and IV) in the TCGA-PAAD cohort.

(D) Relative expression levels of ATP7B across pathological grades (G1, G2, G3, G4, and GX) in the TCGA-PAAD cohort.

(E) Relative expression levels of ATP7B in the TCGA-PAAD cohort stratified by clinical responses to gemcitabine (left) or radiation therapy (right).

(F) Relative expression levels of ATP7B across different age groups (≤65 years vs. >65 years) in the TCGA-PAAD cohort.

(G) Relative expression levels of ATP7B across genders (FEMALE vs. MALE) in the TCGA-PAAD cohort.

(H) Relative cell viability of Patu-8988t cells treated with increasing concentrations of copper chloride (CuCl_2_, 0-200 μM) for 24 h.

(I) Protein expression levels of ATP7B in Patu-8988t cells treated with the indicated concentrations of copper chloride (CuCl_2_,0, 5, 10, and 25 μM) for 24 h, with relative protein expression represented by bar graphs on the right.

(J) Relative mRNA expression of ATP7B in Patu-8988t cells treated with the indicated concentrations of copper chloride (CuCl_2_, 0, 5, 10, and 25 μM) for 24 h.

Data are presented as mean ± SD. **P* < 0.05, ***P* < 0.01, ****P* < 0.001; ns, *P* > 0.05, not significant.

**Figure S2. Silencing ATP7B enhances cuproptosis sensitivity *in vitro***

(A) Cell viability of ASPC-1 cells treated with increasing concentrations of Elesclomol (ES, 0-1000 nM) for 24 h.

(B) Relative cell viability of control (shCtrl) and ATP7B-knockdown (shATP7B) ASPC-1 cells treated with Elesclomol (ES, 0, 100, 200, and 500 nM) for 24 h.

(C) Protein levels of ATP7B and FDX1 in control (shCtrl) and ATP7B-knockdown (shATP7B) cells treated with or without Elesclomol (ES, 200 nM) for 24 h, with relative protein expression represented by bar graphs on the right.

(D) DLAT oligomerization in control (shCtrl) and ATP7B-knockdown (shATP7B) cells treated with or without Elesclomol (ES, 200 nM) for 24 h.

(E) DLAT aggregation and subcellular localization in control (shCtrl) and ATP7B-knockdown (shATP7B) cells treated with or without Elesclomol (ES, 200 nM) for 24 h, with relative fluorescence intensity represented by a bar graph on the right. Scale bar: 10 μm.

(F) Protein levels of ATP7A, FDX1, ATP7B, lipoylated DLAT, lipoylated DLST, and SLC31A1 in Patu-8988t cells transfected with shNC, shATP7A, shATP7B, or dual shATP7A+shATP7B, with relative protein expression represented by bar graphs.

Data are presented as mean ± SD. **P* < 0.05, ***P* < 0.01, ****P* < 0.001; ns, not significant.

**Figure S3. Autophagy activation correlates with lower ATP7B levels**

(A) Correlation analysis between autophagy score and cuproptosis score in the TCGA-PAAD database.

(B) Comparison of ATP7B expression in autophagy-low and autophagy-high groups in the GSE62165 dataset.

(C) qRT-PCR analysis of ATP7B mRNA levels in ASPC-1 cells cultured under different autophagy-inducing conditions (SFM, LG, and EBSS) for 24 h.

(D) Relative levels of intracellular copper, GSH, and SOD activity in ASPC-1 cells after treatment with indicated conditions for 24 h.

(E) DLAT cytoplasmic aggregation and subcellular localization in ASPC-1 cells under control, SFM, LG, or EBSS conditions for 24 h, with relative fluorescence intensity represented by a bar graph on the right. Scale bar: 10 μm.

Data are presented as mean ± SD. **P* < 0.05, ***P* < 0.01, ****P* < 0.001.

**Figure S4.** **Screening of autophagy cargo receptors responsible for selective autophagic clearance of ATP7B**

(A) Protein levels of ATP7B in ASPC-1 cells treated with the proteasome inhibitor MG132 at the indicated concentrations (0, 5, 10, 20, and 40 μM) for 24 h (left), or over different time points (0, 3, 6, 9, and 12 h) at 20 μM (right), with corresponding relative protein quantification represented by bar graphs.

(B) AlphaFold3-based docking score ranking of candidate autophagy cargo receptors with ATP7B.

(C) Protein levels of ATP7B and TAX1BP1 following siRNA-mediated depletion of TAX1BP1 in ASPC-1 cells, with relative protein expression represented by bar graphs.

(D) Co-IP assay assessing ATP7B–TAX1BP1 binding in HEK293T cells, including a UBA-domain–deleted TAX1BP1 mutant to evaluate domain requirement for interaction.

(E) Protein levels of NBR1, p62, and ATP7B in ASPC-1 cells under individual or combined knockdown of NBR1 and p62, with relative protein expression represented by bar graphs on the right.

(F) Relative mRNA levels of ATP7B in ASPC-1 cells upon treatment with autophagy inhibitors (3-MA or NH_4_Cl) for 24 h.

Data are presented as mean ± SD. **P* < 0.05, ***P* < 0.01, ****P* < 0.001; ns, not significant.

**Figure S5. Bioinformatics identification of ZBTB10 as an autophagy-associated prognostic regulator linked to cuproptosis and ATP7B expression.**

(A) Volcano plot illustrating differentially expressed genes between PDAC and normal tissues (padj < 0.05) in the GSE62165 database, with ZBTB10 highlighted.

(B) Kaplan–Meier survival analysis stratifying patients into ZBTB10-high and ZBTB10-low groups in the TCGA-PAAD database, with sample size and at-risk numbers displayed.

(C) Volcano plot showing genes differentially expressed between autophagy-high and autophagy-low cohorts (padj < 0.05) in the TCGA-PAAD database, indicating the distribution of ZBTB10.

(D) Linear regression analysis evaluating the correlation between ZBTB10 expression and cuproptosis score in the GSE57495 database.

(E) Linear regression analysis assessing the association between ZBTB10 and ATP7B expression in the GSE17891 database.

**Figure S6. ZBTB10 governs K63-linked ubiquitination and stability of ATP7B**

(A) Venn diagram showing the integrated screening strategy for ATP7B-associated regulatory candidates and identification of ZBTB10.

(B-C) Relative mRNA levels (B) and protein levels (C) of ZBTB10 expression in paired PDAC tissues and adjacent normal tissues.

(D) Computational docking scores for ATP7B–ZBTB10 binding predicted by AlphaFold-derived models and GRAMM analysis.

(E) Structural model of the ATP7B–ZBTB10 complex.

(F) Co-IP assays assessing the interaction between ZBTB10 and ATP7B in HEK293T cells.

(G) Co-IP analysis evaluating K63-linked ubiquitination of ATP7B in the presence of ZBTB10.

(H-I) Protein stability of ATP7B following ZBTB10 knockdown (H) or ZBTB10 overexpression (I) analyzed under CHX treatment for 0, 12, and 24 h, with relative protein stability quantified on the right.

Data are presented as mean ± SD. ****P* < 0.001.

**Figure S7. ZBTB10 depletion stabilizes ATP7B by reducing its K63-linked ubiquitination**

(A) Protein levels of ZBTB10 in cells transfected with control knockdown (shNC), ZBTB10 knockdown (shZBTB10), or ZBTB10 rescue (overexpression), with relative protein expression represented by a bar graph on the right.

(B) Protein levels of ATP7B in cells transfected with shNC, shZBTB10, or shZBTB10 in combination with ZBTB10 rescue, with relative protein expression represented by a bar graph on the right.

(C) K63-linked ubiquitination status of ATP7B in cells following ZBTB10 depletion and subsequent rescue, with corresponding input controls shown at the bottom.

Data are presented as mean ± SD. ***P* < 0.01, ****P* < 0.001.

**Figure S8 ZBTB10 facilitates NBR1-dependent recognition and autophagic degradation of ATP7B**

(A) Immunoblot analysis of ATP7B and ZBTB10 expression in ASPC-1 and Patu-8988t cells following ZBTB10 overexpression, with relative protein expression represented by bar graphs.

(B) Co-IP of ATP7B with ZBTB10 in HEK293T cells under EBSS treatment for 24 h, followed by immunoblotting with indicated antibodies.

(C) Immunoblot detection of ATP7B levels in the presence or absence of ZBTB10 under EBSS stimulation for 24 h, with relative protein abundance represented by bar graphs on the right.

(D) Co-IP analysis of K63-linked ubiquitination of ATP7B in HEK293T cells transfected with HA-ZBTB10, Flag-ATP7B, and Myc-Ub-K63, with or without autophagy inhibition (3-MA) for 24 h.

(E) Co-IP assessment of ATP7B interaction with NBR1 in the presence of ZBTB10, followed by immunoblot detection of the indicated proteins.

(F) Immunoblot analysis of ATP7B levels in HEK293T cells transfected with HA-ZBTB10 and siRNA targeting NBR1, with relative protein expression represented by bar graphs.

(G) Immunoblot analysis of ATP7B in cells overexpressing wild-type (WT) NBR1 or UBA-deficient NBR1 (ΔUBA), with or without ZBTB10 expression, with relative protein expression represented by bar graphs.

(H) Immunoblot analysis of ATP7B levels in cells overexpressing Flag-ZBTB10 with or without ammonium chloride (NH₄Cl) treatment for 24 h, with corresponding relative protein abundance represented by bar graphs.

Data are presented as mean ± SD. **P* < 0.05, ***P* < 0.01, ****P* < 0.001; ns, not significant.

**Figure S9. Selectivity evaluation of ATAUTAC against related copper homeostatic proteins**

(A) Protein levels of ATP7B in cells treated with increasing concentrations of ATAUTAC (0, 5, 10, 20, 30, and 50μM) for 24 h.

(B) Protein levels of key copper transporters ATP7A and SLC31A1 in cells treated with the indicated concentrations of ATAUTAC (0, 5, 10, 20, 30, and 50 μM) for 24 h, with corresponding relative protein expression represented by bar graphs on the right.

(C) Relative protein levels of copper chaperones ATOX1 and CCS in cells treated with the indicated concentrations of ATAUTAC (0, 5, 10, 20, 30, and 50 μM) for 24 h.

Data are presented as mean ± SD. ****P* < 0.001; ns, not significant.

**Figure S10.** **ATAUTAC induces copper-dependent metabolic stress and suppresses ASPC-1 cell proliferation in a dose-dependent manner**

(A) Relative levels of cellular metabolites, including α-ketoglutarate, pyruvate, succinate, and ATP, in ASPC-1 cells treated with increasing concentrations of ATAUTAC (0, 10, 20, 30, and 50 μM) for 24 h.

(B) Relative levels of intracellular copper, GSH, and SOD activity in ASPC-1 cells treated with the indicated concentrations of ATAUTAC for 24 h.

(C) Representative copper salt staining images in ASPC-1 cells under increasing ATAUTAC concentrations for 24 h. Scale bar: 50 μm.

(D-E) Protein levels of FDX1, ATP7B, lipoylated DLAT, lipoylated DLST, and SLC31A1 in cells treated with the indicated concentrations of ATAUTAC for 24 h (D), with relative protein expression represented by bar graphs in (D, E).

(F) DLAT cytoplasmic aggregation and subcellular localization in ASPC-1 cells treated with the indicated concentrations of ATAUTAC for 24 h, with relative fluorescence intensity represented by a bar graph on the right. Scale bar: 10 μm.

(G-H) Representative immunofluorescence images of mitochondrial ROS (MitoSOX) in ASPC-1 cells exposed to the indicated ATAUTAC concentrations for 24 h (G), with relative MitoSOX Red fluorescence quantified on the right in (H). Scale bar: 5 μm.

(I) Representative EdU staining images showing proliferative activity of ASPC-1 cells treated with the indicated concentrations of ATAUTAC for 24 h, with corresponding quantification of the percentage of EdU-positive cells. Scale bar: 50 μm.

(J) Colony-forming assays of ASPC-1 cells cultured with the indicated concentrations of ATAUTAC for 24 h, followed by standard culture, with corresponding quantification shown on the right.

(K) Relative cell viability of ASPC-1 cells treated with the indicated concentrations of ATAUTAC for 24 h.

Data are presented as mean ± SD. **P* < 0.05, ***P* < 0.01, ****P* < 0.001.

**Figure S11. ATAUTAC induces copper-dependent metabolic stress and suppresses** **Panc02 cell proliferation in a dose-dependent manner**

(A) Relative levels of cellular metabolites, including α-ketoglutarate, pyruvate, succinate, and ATP, in Panc02 cells treated with increasing concentrations of ATAUTAC (0, 10, 20, 30, and 50 μM) for 24 h.

(B) Relative levels of intracellular copper, GSH, and SOD activity in Panc02 cells treated with the indicated concentrations of ATAUTAC for 24 h.

(C) Representative copper salt staining images in Panc02 cells under increasing ATAUTAC concentrations for 24 h. Scale bar: 50 μm.

(D-E) Protein levels of FDX1, ATP7B, lipoylated DLAT, lipoylated DLST, and SLC31A1 in Panc02 cells treated with the indicated concentrations of ATAUTAC for 24 h (D), with relative protein expression represented by bar graphs in (D, E).

(F) DLAT cytoplasmic aggregation and subcellular localization in Panc02 cells treated with the indicated concentrations of ATAUTAC for 24 h, with relative fluorescence intensity represented by a bar graph on the right. Scale bar: 10 μm.

(G-H) Representative immunofluorescence images of mitochondrial ROS (MitoSOX) in Panc02 cells exposed to the indicated ATAUTAC concentrations for 24 h (G), with relative MitoSOX Red fluorescence quantified on the right in (H). Scale bar: 5 μm.

(I) Colony-forming assays of Panc02 cells cultured with the indicated concentrations of ATAUTAC for 24 h, followed by standard culture, with corresponding quantification shown on the right.

(J) Relative cell viability of Panc02 cells treated with the indicated concentrations of ATAUTAC for 24 h.

Data are presented as mean ± SD. **P* < 0.05, ***P* < 0.01, ****P* < 0.001.

**Figure S12. Evaluation of ATAUTAC safety and systemic toxicity *in vivo***

(A) Representative H&E staining images of major organs (heart, liver, spleen, lung, and kidney) collected from tumor-bearing mice treated with vehicle, 20 mg/kg ATAUTAC, or 40 mg/kg ATAUTAC. Scale bar: 100 μm.

(B) Dynamic monitoring of body weight in mice receiving intratumoral administration of ATAUTAC at 20 mg/kg or 40 mg/kg.

(C) Serum biochemical analyses of liver and renal function, including ALT, AST, AST/ALT ratio, and creatinine (CREA), in mice under the indicated treatment conditions.

Data are presented as mean ± SD. ns, not significant.

**Figure S13. ATAUTAC synergizes with GEM to enhance cuproptosis in PDAC cells**

(A) Cell viability assays of ASPC-1 and Patu-8988t cells treated with indicated concentrations of GEM (GEM, 0–10 μM) in the presence or absence of ATAUTAC (50 μM), or exposed to increasing doses of ATAUTAC (0–50 μM) with/without GEM (5 μM) for 24 h.

(B) Representative colony-formation images and quantitative analysis of ASPC-1 and Patu-8988t cells subjected to single or combined treatment with GEM (5 μM) and ATAUTAC (50 μM) for 24 h, followed by standard culture, with relative colony formation quantified on the right.

(C) Representative copper salt staining images of ASPC-1 and Patu-8988t cells under PBS, GEM (5 μM), ATAUTAC (50 μM), or GEM+ATAUTAC treatment conditions for 24 h. Scale bar: 50 μm.

Data are presented as mean ± SD. **P* < 0.05, ***P* < 0.01, ****P* < 0.001.

**Figure S14. Evaluation of systemic safety in mice following ATAUTAC and GEM treatment**

(A) Organ weights of heart, lung, liver, spleen, and kidney collected from mice receiving PBS, GEM (40 mg/kg), ATAUTAC (40 mg/kg), or combination GEM+ATAUTAC treatment (n = 5 mice per group).

(B) Longitudinal monitoring of mouse body weight during the 30-day therapeutic course under the indicated treatment conditions (n = 5 mice per group).

(C) Serum biochemical levels, including ALT, AST, AST/ALT ratio, and CREA, in mice from each treatment group at the experimental endpoint (n = 5 mice per group).
